# The Second Wave of COVID-19 in South and Southeast Asia and the Effects of Vaccination

**DOI:** 10.3389/fmed.2021.773110

**Published:** 2021-12-14

**Authors:** Haitao Song, Guihong Fan, Yuan Liu, Xueying Wang, Daihai He

**Affiliations:** ^1^Complex Systems Research Center, Shanxi University, Taiyuan, China; ^2^Department of Mathematics, Columbus State University, Columbus, OH, United States; ^3^Department of Applied Mathematics, Hong Kong Polytechnic University, Kowloon, Hong Kong SAR, China; ^4^Department of Mathematics and Statistics, Washington State University, Pullman, WA, United States

**Keywords:** COVID-19, Southeast Asia, South Asia, mathematical modeling, Delta variants, vaccination, infection attack rate, infection fatality rate

## Abstract

**Background:** By February 2021, the overall impact of coronavirus disease 2019 (COVID-19) in South and Southeast Asia was relatively mild. Surprisingly, in early April 2021, the second wave significantly impacted the population and garnered widespread international attention.

**Methods:** This study focused on the nine countries with the highest cumulative deaths from the disease as of August 17, 2021. We look at COVID-19 transmission dynamics in South and Southeast Asia using the reported death data, which fits a mathematical model with a time-varying transmission rate.

**Results:** We estimated the transmission rate, infection fatality rate (IFR), infection attack rate (IAR), and the effects of vaccination in the nine countries in South and Southeast Asia. Our study suggested that the IAR is still low in most countries, and increased vaccination is required to prevent future waves.

**Conclusion:** Implementing non-pharmacological interventions (NPIs) could have helped South and Southeast Asia keep COVID-19 under control in 2020, as demonstrated in our estimated low-transmission rate. We believe that the emergence of the new Delta variant, social unrest, and migrant workers could have triggered the second wave of COVID-19.

## Introduction

Coronavirus disease 2019 (COVID-2019) (caused by SARS-CoV-2) invaded the world unexpectedly in 2019 and changed human life tremendously (1). The SARS-CoV-2 virus is transmitted *via* respiratory droplets with an incubation period of 2–14 days (2). Common symptoms of light infection include fever, cough, and shortness of breath, while severe infections may require intensive care or ventilator support (2). Ever since the WHO declared the disease a pandemic on March 11, 2020 (3), there have been 199.67 million confirmed cases worldwide, and more than 4 million deaths were reported (4) as of August 4, 2021.

**Figure 1:**
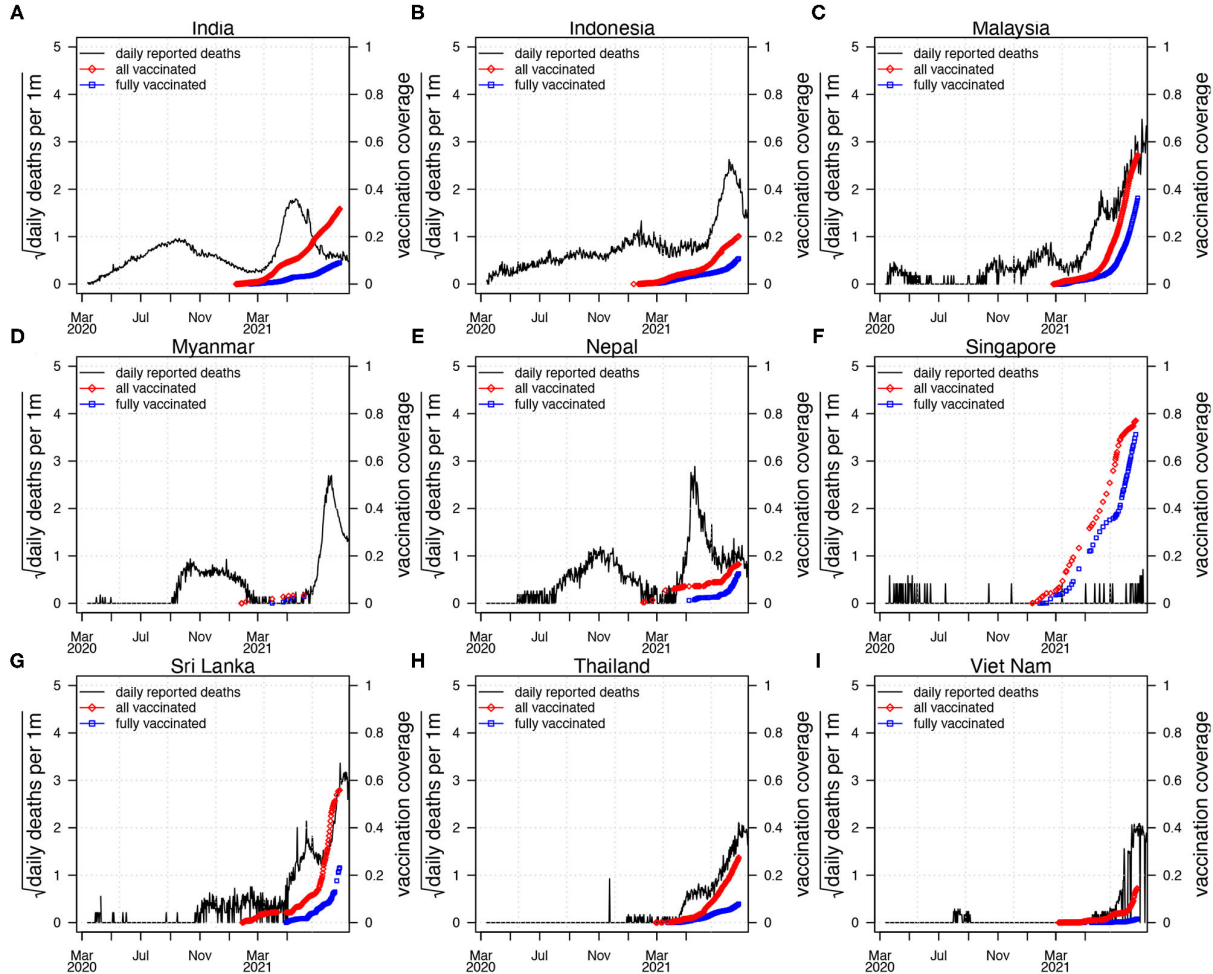
Reported confirmed coronavirus disease 2019 (COVID-19) deaths (black curve) in nine countries in SEA and SA, with vaccination coverage. All the vaccinated proportion (red diamond, vaccinated/one plus dose) and fully vaccinated proportion of the population (blue square, fully vaccinated). The first wave of COVID-19 epidemic occurred largely before March 2021, and the second wave of COVID-19 epidemic appeared after March 2021. **(A)** India, **(B)** Indonesia, **(C)** Malaysia, **(D)** Myanmar, **(E)** Nepal, **(F)** Singapore, **(G)** Sri Lanka, **(H)** Thailand, **(I)** Viet Nam.

**Figure 2:**
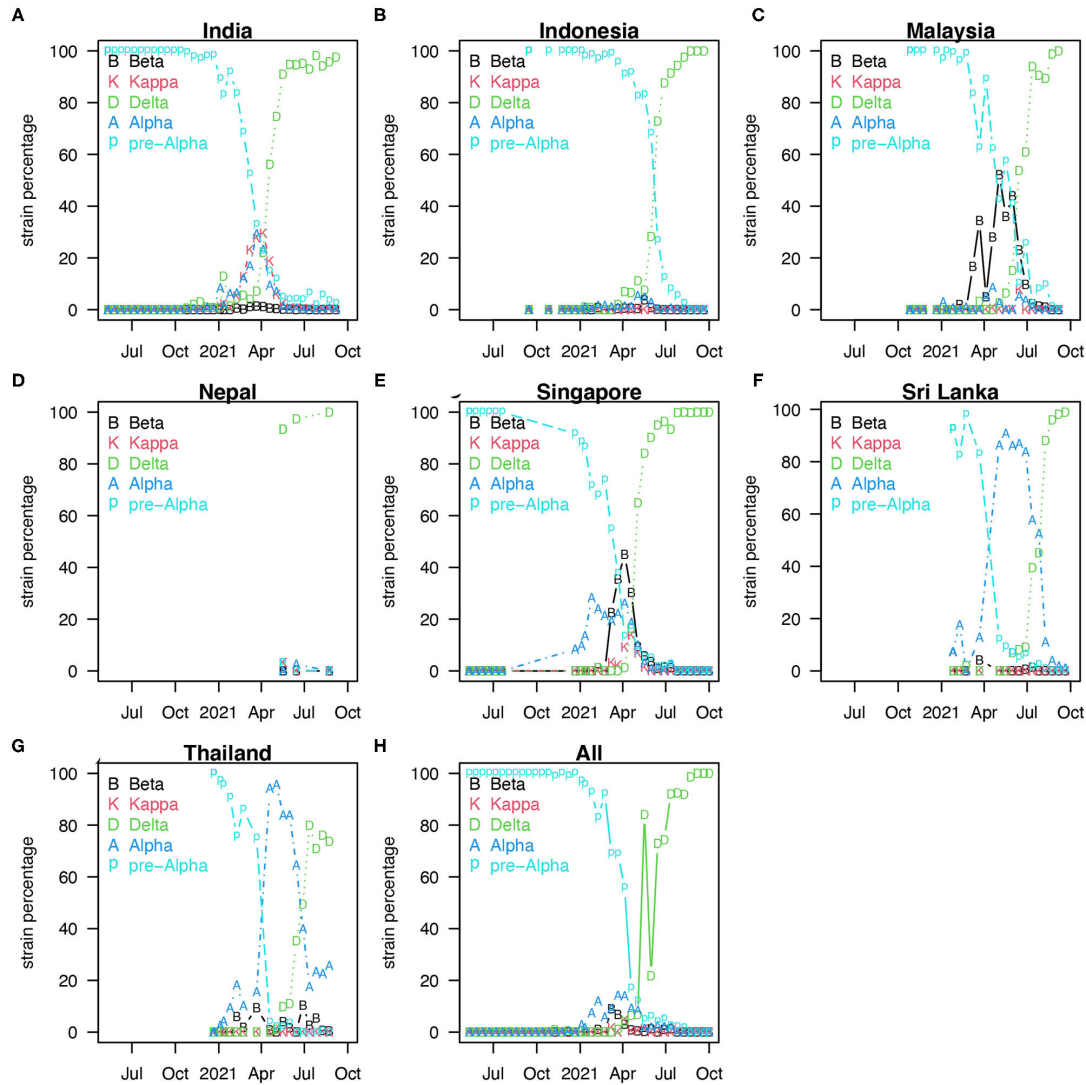
Percentage of strains sequenced biweekly [strain percentage in seven countries of SA and SEA **(A–G)]** where variants sequenced data are available (8). We only show these strains with a maximum percentage > 20%. **(H)** Showed the median of strain percentage for each strain sequenced in the seven countries.

Coronavirus disease 2019 pandemic in South Asia (SA) and Southeast Asia (SEA) was mild in 2020 compared with other hot spots such as Europe or North America (see Figure 1). Figure 1 shows the reported COVID-19 deaths in nine countries of SA and SEA, with the vaccination coverage. However, the appearance of the new variants of concern (VOC) changed the nature of the pandemic dramatically in SA and SEA with a sudden increase of cases in many countries in this region (5). In Singapore, Vietnam, and Malaysia, COVID-19 was well-controlled at the earlier pandemic stages with both non-pharmacological interventions (NPI) (5), social media (6, 7), and vaccination. The emergence of new VOC has reversed this trend and caused a new wave of infection. Since April 2021, SA and SEA have been plagued by a new, more contagious Delta variant of COVID-19, which was the first reported in India in October 2020 (6) (see Figure 2). According to (6), the new variant was one of the main factors that accounted for the ongoing wave of COVID-19 in SA and SEA. In Figure 2, we show the strain (pre-Alpha strain and new variants) percentage sequenced in seven countries of SA and SEA where such data are available. We only show strain which had a maximum percentage > 20%. The main picture is that the Delta variant replaced the pre-Alpha strain directly, while Alpha and Beta strains only dominated shortly in some of the seven countries.

The Delta variant caused an increasing crisis in Indonesia and other nearby regions (7, 9) (see Figures 1, 2). By August 18, 2021, SEA has been fighting with the highest COVID-19 death toll of the world caused by VOC, but enhanced by the relatively low coverage of vaccines (10). Based on the survey (11) by the Indian Council of Medical Research (ICMR), around 21.4% of 28,589 adolescent participants had been infected as of February 4, 2021. Another serological study (12) found that the prevalence of IgG antibody (indication of past infection) to SARS-CoV-2 was 11.4% in East Java, Indonesia. A serological study (13) among healthcare workers in the Kathmandu valley, Nepal, showed a 23% positivity for polymerase chain reaction (PCR) testing. SARS-CoV-2 seroprevalence study (14) indicated that a symptom-based PCR-testing strategy missed 62% of COVID-19 diagnoses, and ~36% of individuals with SARS-CoV-2 infection were asymptomatic. These serological studies found a much higher infection attack rate (IAR, proportion of the infected population) than the estimated IAR based on reported cases. Therefore, these serological studies should be considered in modeling if a reasonable IAR estimation and useful forecast for the pandemic is expected.

In Table 1, the survey in India involved 70 districts across India. Each district recruited 400 members of the general population (over 10 years old) and 100 healthcare workers. After adjustment for weighting and assay characteristics, for 28,598 serum samples, the seropositive rate of antibodies against either N protein or S1 receptor-binding domain protein was 24.1% (15).

**Table 1:** Coronavirus disease 2019 (COVID-19) serum survey conducted in three places.

**Time**	**Place**	**People**	**Blood serum**
2020/12–2021/01	India	28,598(general population and healthcare workers (HCWs))	Antibodies 24.1% with 23.0%−25.3% (95% CI)
2020/06–2020/12	East Java, Indonesia	1,819	IgG antibody to SARS-CoV-2 was 11.4% (207/1,819)
2020/03/25–2020/07/25	Singapore	198,320 migrant workers	111,280 residents with a positive PCR or serology result, for an overall infection prevalence of 56.1% with 55.9–56.3% (95% CI)

Because of the limited data on COVID-19 prevalence in Indonesia, there was an investigation into SARS-COV-2, which causes COVID-19 disease. From June to December 2020, 1,819 participants aged 16 years or older were recruited from Surabaya city. An overall 11.4% prevalence of IgG antibodies against SARS-COV-2 was observed in the subjects (see Table 1). Using a chi-squared test of categorical variables, there is a significant difference in the prevalence of SARS-COV-2 antibodies between working and occupational groups. A higher prevalence of IgG was detected in laboratory technicians (22.2%) compared with healthcare workers (6.0%) directly treating patients with COVID-19 and other healthcare workers (2.9%) (12).

As a part of the national public health response to COVID-19, PCR and serum tests were conducted on migrant workers residing in all dedicated hostels in Singapore between March 25 and July 25, 2020. This included 43 dormitories with a total population of 198,320 (63.6% underwent PCR testing, and 68.4% underwent serological testing). PCR or serological results were positive in 111,280 inhabitants, with an overall infection rate of 56.1% (and 55.9–56.3% of 95% CI) (16) (see Table 1).

## Materials and Methods

Mathematical modeling has been successfully used to forecast COVID-19 trends and has assisted public health organizations in policy making. This article uses a simple compartmental epidemic model. Our model considers a time-dependent transmission rate since both changes inhuman behavior, and the use of control measures can alter the transmission rate of the disease. Other epidemiological parameters such as the latency period and the infection fatality rate (IFR) may vary as well. To simplify the model, we focus only on the time-varying transmission rate and assume that other parameters are constant.

The population is divided into susceptible (*S*), exposed (*E*), infectious (*I*), hospitalized or delayed (*T*), death (*D*), and recovered/immunized classes (*R*), respectively. Susceptible individuals can become exposed after getting contact with an infectious people. Exposed individuals can become infectious after the latency period. Infectious individuals can move to the hospitalized (or delayed) classes and recovered/immunized classes. Hospitalized (or delayed) individuals may die or survive and recover/be immunized. In addition, recovered/immunized individuals become susceptible after vaccine immunization failure. We choose the model and parameter values from (11, 17, 18), with the addition of vaccination.


$$\begin{array}{r} Ṡ=-\beta SI-\eta ṽ\left(t-\tau \right)S\\ Ė=\beta SI-\sigma E\\ İ=\sigma E-\gamma I\\ Ṫ=\phi \gamma I-\kappa T\\ Ḋ=\theta \kappa T\\ Ṙ=\left(1-\phi \right)\gamma I+\left(1-\theta \right)\kappa T+\eta ṽ\left(t-\tau \right)S \end{array}$$

Here, *S*, E, *I*, and *R* are susceptible, exposed, infectious, and recovered/immunized classes, respectively. *T* denotes a delay class between infectious and death classes, while *D* denotes death class. β denotes a time-varying transmission rate which is modeled using an exponential cubic spline (19–21) with a fixed number of notes (*n*_β_ = 9) evenly cover the study period. Parameters σ, γ, and κ are rates at which the individual loses exposed status and infectious status, σ = 0.5 days, γ = 1/3 days, and κ = 1/12 days. Thus, we have a mean generation time (σ^−1^ + γ^−1^) of 5 days and a mean delay between loss of infectiousness to death 12 days that are well in line with estimates in previous studies (22). The IFR is φθ. One cannot estimate simultaneously ϕ and θ with only the death data. Thus, we choose to fix ϕ and estimate θ. Parameter ṽ is the vaccination rate per capita among unvaccinated. The rate at which susceptible individuals get vaccinated is ṽ(*t*) = $v(t)/(1-\int_{0}^{t-1}v(s)ds)$, where *v*(*s*) represent the vaccination rate per capita among all population per day. When we incorporated this effective vaccination rate, we considered a delay of 2 weeks (τ) for the vaccine to take effect and uniform vaccine efficacy of η =85%. We note that this is the simplest model for this situation. Given that seven of the nine countries have a vaccination coverage (fully vaccinated) of less than 20%, the effect of the vaccination in these seven countries would be mild. In Malaysia and Singapore, especially in Singapore, the vaccination coverage is high, and we first fit the model with the vaccination, then we simulated our fitted model without vaccination. Thus, we showed the effects of the vaccination campaign. We used the famous R package POMP. A detailed description of the usage of this package on epidemiological models can be found here (23–25). We provide sensitivity analyses in the Supplementary Material, where we consider different number of nodes in the transmission rate (*n*_β_ = 8), a lower vaccine efficacy (η =75%), and different model structure (i.e., with an explicitly vaccinated class with a reduced susceptibility) and reduced IFR due to vaccination.

We fit a unified model to the reported COVID-19 deaths in nine countries in this region. Our study period is from March 11, 2020 to August 17, 2021.We assume the transmission rate to be an exponential cubic spline function spanning the whole time series. This semi mechanistic approach of modeling multiple waves of infections has been successfully used in the previous epidemics and pandemics.

## Results

Using data from the WHO dashboard, Figure 1 shows the confirmed COVID-19 deaths and vaccination coverage (including partly vaccinated and fully vaccinated) of the nine countries (India, Indonesia, Malaysia, Myanmar, Nepal, Singapore, Sri Lanka, Thailand, and Vietnam). These nine countries account for most deaths in SA and SEA (26). Figure 1 shows that the first wave of COVID-19 epidemic remains relatively low, while the second wave causes a much higher level of COVID-19 pandemic when specific vaccine coverage is achieved.

Figure 2 shows the percentage of strains sequenced biweekly (strain percentage) in the seven countries of SA and SEA where variants sequenced data are available (8). Panel h shows the median of strain percentage for each strain sequenced in the seven countries. The overall strain-dominance pattern in SA and SEA is that Delta strain replaced pre-Alpha directly, while Alpha and Beta strains only dominated in some among the seven countries but not all.

**Figure 3:**
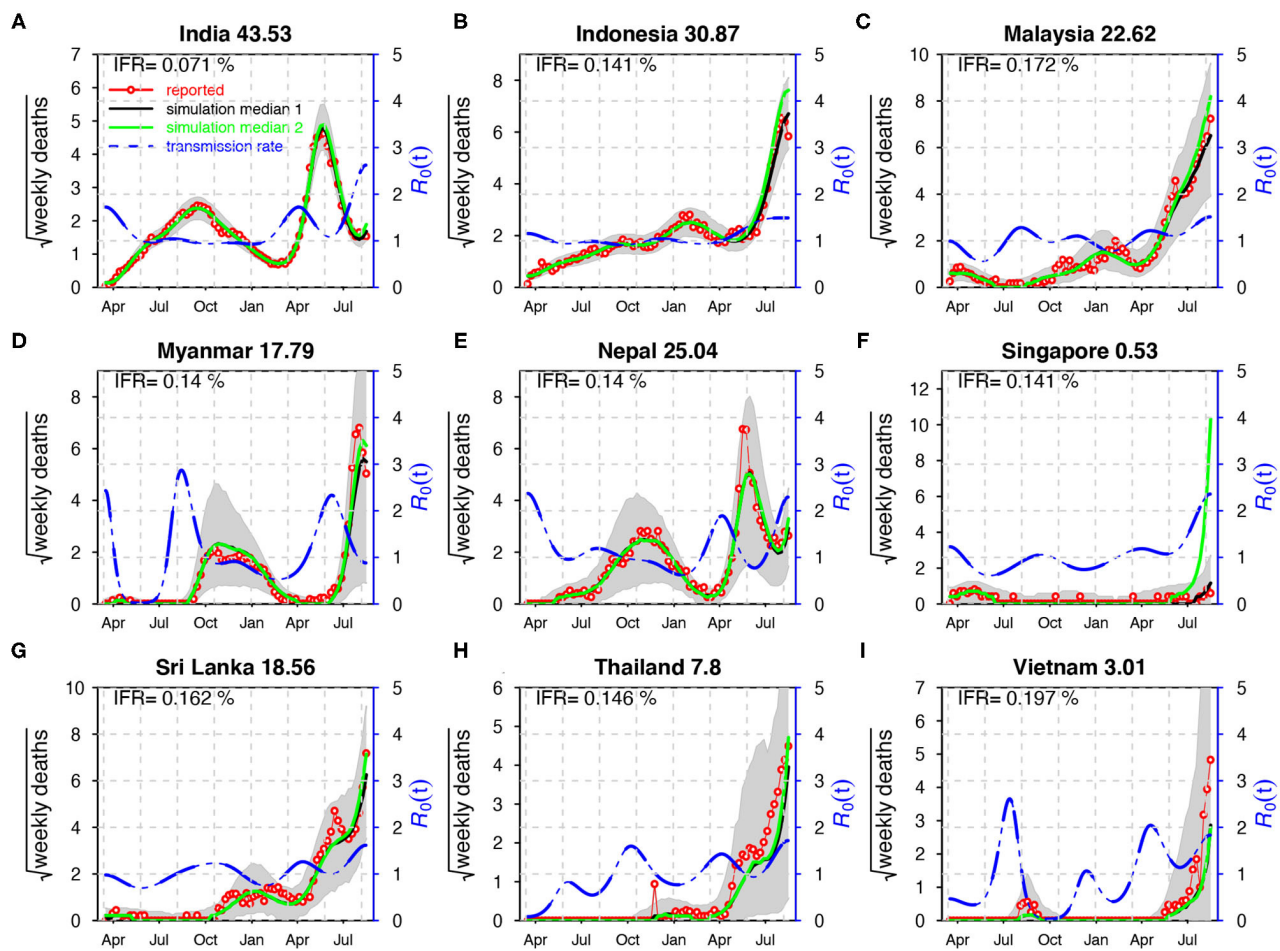
Fitting the model to the reported deaths in India **(A)**, Indonesia **(B)**, Malaysia **(C)**, Myanmar **(D)**, Nepal **(E)**, Singapore **(F)**, Sri Lanka **(G)**, Thailand **(H)**, and Viet Nam **(I)** with a time-varying transmission rate. The red circles denote the daily reported COVID-19 deaths. The black curve denotes the median of 1,000 model simulations with vaccination, while the green curve shows the simulation median without vaccination. The shaded region denotes the 95% CI of the 1,000 model simulations. The blue-dashed curve denotes the reconstructed transmission rate. ϕ = 0.008 for India; ϕ = 0.03 for other countries. Here, $R_{0}=\beta \left(t\right)/\gamma$. The IFR is shown in each panel and the IAR is shown above each panel.

In Figure 3, the SEITDR (Susceptible-Exposed-Infectious-Hospitalized-Death-Recovered/immunized) model simulated the reported deaths from COVID-19 for the nine countries in SA and SEA and investigated the dynamics of COVID-19 in this region. The fitting results were used to evaluate the time-varying transmission rate [in terms of reproduction number $R_{0}\left(t\right)$] for each country. From Figure 3, except for Singapore, all other eight countries shared a similar pattern: a lower level of the first wave and a higher level of the second wave of infection of COVID-19. Our model simulation well-matched the reported deaths in the nine countries. The IFR is similar in most of the countries at a level of 0.21% except for India at a level of 0.071%. The transmission rate varied dramatically in some countries, likely due to invasion of new variants and on–off of control measures. The red circles are the daily reported deaths; the black curve denotes the median of 1,000 model simulations; the shaded region denotes the 95% CI of the 1,000 simulations. The green curve shows the simulation median without vaccination while other parameters were kept. The blue-dashed curve denotes the reconstructed time-varying transmission rate. The deviation of the green and the black curves illustrates the effects of the vaccination. Results of sensitivity analyses are provided in Supplementary Figures S1–S4. We found that our results in the main text are robust to a variety of parameter and model modifications.

## Discussion and Conclusion

Ever since the emergence of COVID-19 in 2019, it has spread worldwide (more than 200 countries and territories) rapidly. Countries in SA and SEA have implemented prevention and mitigation strategies to combat COVID-19 pandemic, including testing, contact tracing, and border control (5). In Table 1, we compare three serum surveys in the three locations. The Indian government-initiated serum surveys indicate a 24.1% positivity of serum antibody (15). An 11.4% overall prevalence of IgG antibodies is demonstrated, but the prevalence of IgG in laboratory technicians was much higher than in other professions in East Java, Indonesia (12). The Singapore government tested all the migrant workers in dedicated hostels through PCR and serological methods within 4 months and obtained an overall infection rate of 56.1% (16).

Figure 1 shows COVID-19 death and vaccination coverage trends in SA and SEA, and demonstrates that COVID-19 epidemic first wave infections remain relatively low without vaccination coverage. In contrast, COVID-19 epidemic second wave infections attained a much higher level, likely due to invasion of the Delta strain (see Figure 2) and possibly relaxing of control measures.

It is well-recognized that because of the implementation of NPIs, COVID-19 pandemic in SA and SEA was kept under control in 2020 (5). Subsequently, a new wave of COVID-19 pandemic has appeared in 2021.The emergence of VOC was believed to be one of the key driving factors for the second wave of COVID-19 in SA and SEA (6). Except for this common factor, the second wave of COVID-19 demonstrates some unique features that vary between countries. Multiple factors (including a lack of nationwide preparations and poorly implemented or enforced health and safety precautions during festivals, sporting events, and state/local elections) could have caused the rapid expansion of the epidemic in India and resulted in a large number of deaths. The test positivity rate increased dramatically from 2% on March 1 to 22% on May 1 during the second wave in India. In Myanmar, a series of events in February 2021 possibly treated national health and favored the spread of the virus (27). The second wave in Nepal worsened possibly due to the returning of many migrant workers from neighboring countries (23). In Thailand, a group of young, urban, upper-middle class individuals got infected after visiting nightclubs and restaurants in Bangkok during the long weekend. Then, the disease spread to their families and relatives and across the whole country quickly (28). All these particular reasons could have caused the second wave of COVID-19 to worsening SA and SEA. The reasons for low IFR estimated here include that we assume that COVID-19 death data were accurate. In reality, deaths were under reported. While the serological studies in these countries suggested that a large proportion of the population had been infected. The IFR may be regarded as a ratio of infection to reported death rather than the true IFR in these countries.

This work has several limitations, including the assumption that all parameters are constant except for the transmission rate and that the model is for the whole country while we ignored the heterogeneity across age groups and regions. This study relies on the reported death data and adopted a non-mechanistic cubic spline type of transmission rate. Alternatively, one could consider explicitly incorporating all kinds of control measures (such as the Google mobility matrix) into their model and expect to get more insightful observations on the transmission status of COVID-19 in SA and SEA. This work should be treated as a simple conceptual modeling attempt to estimate the size of the epidemic (IAR) in this region and the associated large-scale trends. Our estimated IAR is essentially in line with serological studies, and we illustrate the effects of vaccination *via* a straightforward approach. The actual IFR and vaccination effect are much more challenging to model. Nevertheless, we have laid the groundwork for further improvement.

Before the vaccine coverage reaches sufficiently high level, public health department must establish and maintain strong public health infrastructure and NPI to respond effectively to combat COVID-19 pandemic in places where the IAR was low and vaccination coverage was high, vaccination saved a lot of lives.

## Data Availability Statement

Publicly available datasets were analyzed in this study. The data are from World Health Organization website.

## Author Contributions

DH, HS, and GF conceived the study, performed data analysis, and wrote the draft. DH, HS, YL, XW, and GF discussed the results, revised the manuscript critically, and approved it for publishing. All authors contributed to the article and approved the submitted version.

## Funding

This study described in this article was partially supported by a grant from the Research Grants Council of the Hong Kong Special Administrative Region, China (HKU C7123-20G), the Fund Program for the Scientific Activities of Selected Returned Overseas Professionals in Shanxi Province (20200001), the National Natural Science Foundation of China (12171291), and the Key Research and Development Project in Shanxi Province (202003D31011/GZ).

## Conflict of Interest

The authors declare that the research was conducted in the absence of any commercial or financial relationships that could be construed as a potential conflict of interest.

## Publisher's Note

All claims expressed in this article are solely those of the authors and do not necessarily represent those of their affiliated organizations, or those of the publisher, the editors and the reviewers. Any product that may be evaluated in this article, or claim that may be made by its manufacturer, is not guaranteed or endorsed by the publisher.
