# Supplementary material for: The Second Wave of COVID-19 in South and Southeast Asia and the Effects of Vaccination
Source: Front Med (Lausanne). 2021 Dec 14;8:773110. doi: 10.3389/fmed.2021.773110 (PMC8712656; doi:10.3389/fmed.2021.773110)
Supplement: Supplementary file 1 [file Data_Sheet_1.docx]

**Supplementary Materials**

**Figure S1.** Reproduction of Figure 3 with a vaccine efficacy $\eta=0.75$, and other parameters are the same as Figure 3.

**Figure S2.** Reproduction of Figure 3 with $n_{\beta}=8$, and other parameters are the same as Figure 3.

**Figure S3**. Reproduction of Figure 3 with an explicit vaccinate class $V$, with $\dot{V}=\left( 1-\eta\right)\tilde{v}(t-\tau)S-\varepsilon\beta VI$, with a reduced susceptibility $\varepsilon=0.75$.

**Figure S4**. Reproduction of Figure S3 with $\varepsilon=0.8$ and a reduced IFR, where $\theta$ with $\tilde{\theta}(t)=(1-\omega\int_{0}^{t} v\left( s \right)ds) \theta(t)$. When the cumulative vaccination coverage approach 100%, the IFR will reduce by $\omega$ proportion. Here we set $\omega=0.25$.

**Figure S4**. Reproduction of Figure S4 with $n_{\beta}=8$, and other parameters are the same as Figure S4.
